# Role of smooth muscle cells in Cardiovascular Disease

**DOI:** 10.7150/ijbs.49871

**Published:** 2020-08-21

**Authors:** Yingzhi Zhuge, Jian Zhang, Fanyu Qian, Zhengwang Wen, Chao Niu, Ke Xu, Hao Ji, Xing Rong, Maoping Chu, Chang Jia

**Affiliations:** 1 Pediatric Research Institute, The Second Affiliated Hospital and Yuying Children's Hospital of Wenzhou Medical University, Wenzhou 325027, China.; 2 Children's Heart Center, Institute of Cardiovascular Development and Translational Medicine, The Second Affiliated Hospital and Yuying children's Hospital of Wenzhou Medical University, Wenzhou 325027, China.; 3 The Institute of Life Sciences, Wenzhou University, Wenzhou, Zhejiang, China.

**Keywords:** smooth muscle cells, cardiovascular disease, phenotype switching, arterial aneurysms

## Abstract

Normally, smooth muscle cells (SMCs) are localized in the tunica media of the vasculature, where they take responsibility for vascular contraction and extracellular matrix (ECM) generation. SMCs also play a significant role in obedience and elastic rebound of the artery in response to the haemodynamic condition. However, under pathological or stressed conditions, phenotype switching from contractile to synthetic state or other cell types will occur in SMCs to positively or negatively contribute to disease progression. Various studies demonstrated that functional changes of SMCs are implicated in several cardiovascular diseases. In this review, we present the function of vascular SMCs (VSMCs) and the involved molecular mechanisms about phenotype switching, and summarize the roles of SMCs in atherosclerosis, hypertension, arterial aneurysms and myocardial infarction, hoping to obtain potential therapeutic targets against cardiovascular disease in the clinical practices.

## Introduction

Muscle cells are active, contractile cells in animals, which are slender in shape, also known as muscle fibers. Unlike all other tissues, muscle cells contain a large number of muscle filaments, which have the characteristics of contractile movement and are the power source for the movement of body organs. Generally speaking, according to the different structure and contraction characteristics, the muscle tissues in human body are divided into three different types as follows: cardiac muscle, skeletal muscle and smooth muscle, among which cardiac muscle and skeletal muscle have alternating horizontal lines under the light microscope, so collectively referred to as striated muscle. Cardiac muscle is composed of cardiomyocytes, which can be divided into working cells and autonomic cells according to their histological and electrophysiological characteristics, and functional differences to maintain the systolic and autonomic activities of the heart. Skeletal muscle, mainly distributed in the limbs, is superior to smooth muscle and forms rapid and powerful contractions. Smooth muscle is composed of smooth muscle cells (SMCs), which are widely distributed in blood vessel walls and many internal organs, and maintain relatively slow and prolonged contraction. In recent years, more and more studies have shown that SMCs are involved in the occurrence and development of various cardiovascular diseases.

## SMCs contribute to smooth muscle contraction

It is well known that smooth muscle is short of transverse striations found in skeletal and cardiac muscle cells, and is often found in the muscular layers of gastrointestinal, respiratory, urogenital, vascular, and lymphatic vessels, as well as in the tunica of some parenchymal organs. The tension contraction of smooth muscle not only resists the gravity or the applied load, but also maintains the normal shape of the organs, and achieves their motor function.

**Figure 1:**
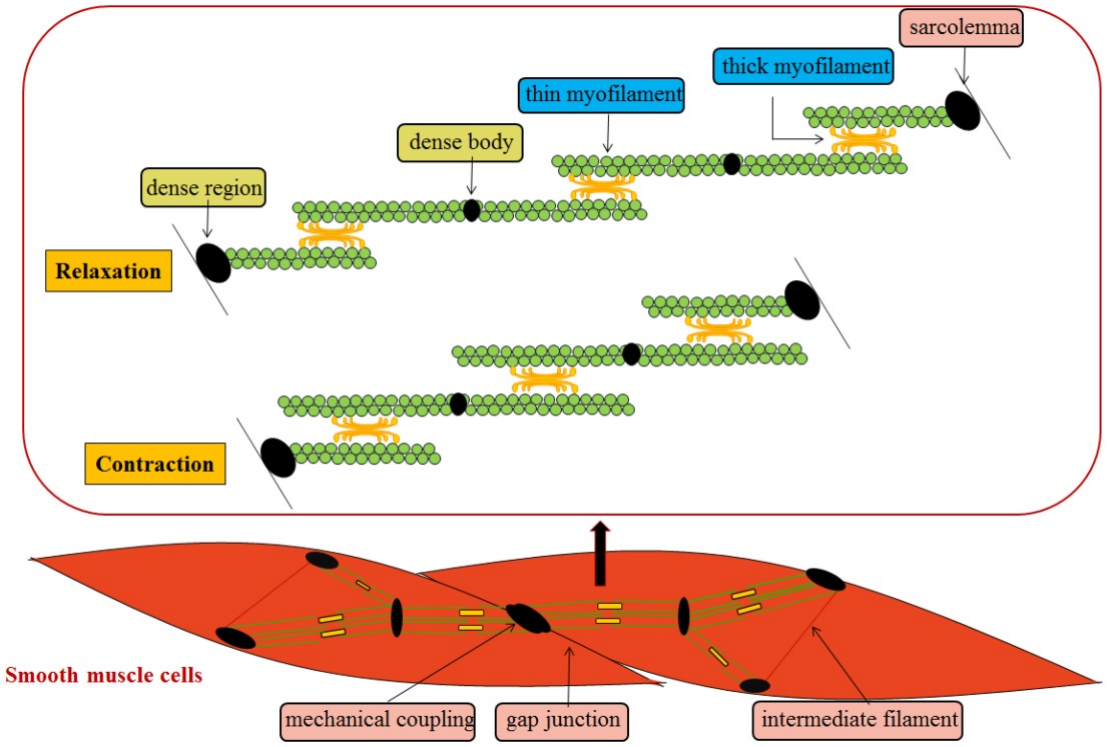
Mechanisms of SMCs contraction and relaxation.

In general, SMCs are slender and spindle-shaped, with a diameter of 1~5 μm and great length variability. It is characterized by the existence of a cytoskeleton inside the cell, which contains some ovoid dense body structures. The spacers appear on the inner side of the cell membrane and become dense regions. The two membranes are closely connected to each other, forming a mechanical coupling to complete the transfer of inter-cell tension. At the same time, gap junctions exist between cells to realize the electric and chemical coupling between cells. Similar protein components to those in the z-band of skeletal muscle are found in the dense body and region, and there is also a filamentous polymer called intermediate filament between the dense body and region, from which the filamentous binding of the dense body and the dense region inside the membrane forms a complete intracellular framework. The thick myofilament of SMCs extend the cross-bridge in different directions in the opposite direction, so that the thin myofilament of different directions slide towards each other, and the sliding range between the thick myofilament and the thin myofilament can extend to the whole length of the thin myofilament, so that the contraction range is larger 1, 2 (**Figure 1**). Compared with skeleton muscle cells, SMCs contract slowly mainly due to that the formation of some longitudinal shape of the pouch concave on cell membrane and absence of embedded T tube result in the increased surface area of the cell membrane, and slower rate to deliver the action potential on the cell membrane.

**Figure 2:**
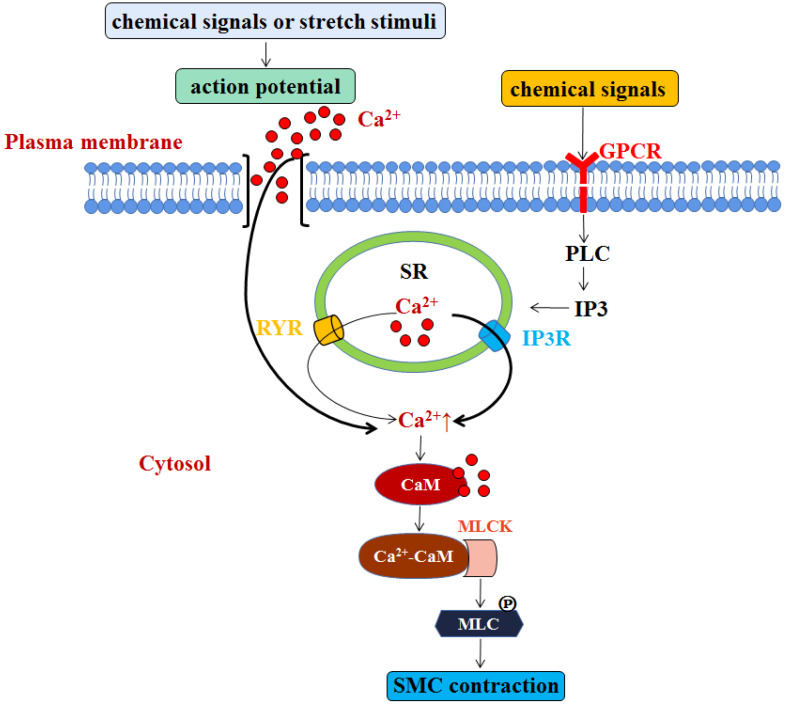
** SMC contraction is modulated by two manners that are both involve Ca^2+^.** One is through action potential induced by chemical signals or stretch stimuli, which promotes extracellular Ca^2+^ influx into intracellular cytosol. Most cytosolic Ca^2+^ comes from extracellular sources, but a small part is released by the RYR. Another is G-protein coupled receptor-PLC-IP3 pathway triggered by chemical signals, which then activates IP3R in the SR membrane to release Ca^2+^ into the cytosol. The elevated level of Ca^2+^ combines with CaM to form Ca^2+^-CaM compounds, which then combine with and activate cytoplasmic myosin light chain kinase (MLCK). The activation of MLCK promotes the phosphorylation of myosin light chain (MLC) for SMC contraction.

We know that Ca^2+^ is the main factor that triggers SMC contraction. Although the sarcoplasmic reticulum (SR) of SMCs is underdeveloped, both Ca^2+^-sensitive ryanodine receptor (RYR) and Inositol 1,4,5-trisphosphate (IP3)-sensitive receptors (IP3R) in the SR membrane act as calcium releasing channels 3. When the cell is activated, Ca^2+^ enters the cell through electric-mechanical coupling and pharmaco-mechanical coupling. In the former manner, action potential is generated by chemical signals or stretch stimuli, which promotes extracellular Ca^2+^ influx into intracellular cytosol. Most cytosolic Ca^2+^ comes from extracellular sources, but a small part is released by the RYR. In the latter manner, chemical signals can activate the G-protein coupled receptor-PLC-IP3 pathway, which then activates IP3R in the SR membrane to release Ca^2+^ into the cytosol 4, 5. SMCs do not contain troponin, but have a calmodulin (CaM). In the majority of SMCs, cytosolic Ca^2+^ concentration is increased, and increased Ca^2+^ combines with CaM to form Ca^2+^-CaM compounds. The compounds then combine with and activate cytoplasmic myosin light chain kinase (MLCK) to promote the phosphorylation of myosin light chain (MLC) for SMC contraction 6 (**Figure 2**). Otherwise, when the smooth muscle cells are relaxed, cytosolic Ca^2+^ will be returned back to SR through the SR membrane calcium pump, or be transported out of the cells through the muscle membrane Na^+^-Ca^2+^ exchanger and calcium pump, making the drop of the cytosolic Ca^2+^ concentration, MLCK deactivation, and the dephosphorylation of phosphorylated MLC by the action of MLC phosphatase (MLCP) in the cytoplasm, eventually resulting in relatively slow relaxation of SMCs 7. Besides, endodermal nitric oxide can also increase the activity of MLCP by regulating cyclic guanosine monophosphate (cGMP) and cyclic adenosine monophosphate (cAMP), and subsequent protein kinase G (PKG) and protein kinase A (PKA), and ultimately achieve SMCs relaxation 8, 9. In addition to calcium-dependent mechanism, vascular contraction is also regulated by calcium-independent mechanisms which related to the alteration of calcium sensitization, the remodeling of actin cytoskeleton, and reactive oxygen species (ROS) 10-12.

## Smooth muscle cell plasticity

SMCs play a key role in controlling blood pressure and blood distribution, and maintaining vascular structural integrity. SMCs are involved in physiological and pathological vascular remodeling by dynamically regulating its phenotypes. Nowadays, there are increasing researches on the phenotype plasticity of SMCs. The contractile and synthetic phenotypes of SMCs are considered as the most extreme ones, while the rest fall in between 13. Under normal condition, SMCs exist as a highly differentiated contractile phenotype. Yet, SMCs turned to de-differentiated proliferative or migratory phenotype under stressed conditions. Therefore, SMCs become difficult to recognize if the expression of their marker proteins is remarkably reduced or completely lost, or the SMCs express marker proteins of macrophages, mesenchymal stem cells, osteochondrogenic cells and myofibroblast-like cells etc. 13, 14.

Several factors can modulate SMC phenotype transition, such as integrins and miRNAs. Integrins are transmembrane receptors that link the cell cytoskeleton to the ECM, which involves in the changes of SMC phenotypes. For example, the highly expressed integrins α1β1 and α7β1 in SMCs can bind to collagen IV and laminin, respectively. Their absence transforms the SMCs to a synthetic epithelioid state from a constrictive and spindle phenotype, and promotes the proliferation and accumulation of SMCs during the thickening of the experimental intima. In contrast, serum α2β1, α5β1 and αvβ3 are highly expressed in synthetic SMCs, which promote collagen deposition, participate in fibronectin deposition, and prevent oxygen-low density lipoprotein (Ox-LDL)-mediated SMC apoptosis to promote plaque stability 15-18. Besides, miRNAs are also particularly involved in regulating phenotype switching of SMCs. Mature miRNA derives from Dicer-cleaved precursor miRNA. The generated miRNA is important for SMC function and development by modulating proliferation and contractile differentiation 19, and also function in the regulation of SMC phenotype. For example, miR-143/145 clusters strongly promote the contractility of the SMC phenotype, while miR-21, miR-221, and miR-222 contribute to the proliferation of SMCs 20, 21.

In recent years, researches on SMC phenotype conversion have been conducted in the following three cases 22: (1) In the process of vascular injury, SMC contraction gene expression is lost. (2) The expression of some classical marker genes used for SMC identification is not limited to SMC lineage, but can also be expressed by other cell types. (3) Cell types that do not express the SMC marker gene under physiological conditions can change to the SMC-like state in diseased or damaged blood vessels.

## Transcriptional and epigenetic modulation of differentiated markers of SMCs

**Figure 3:**
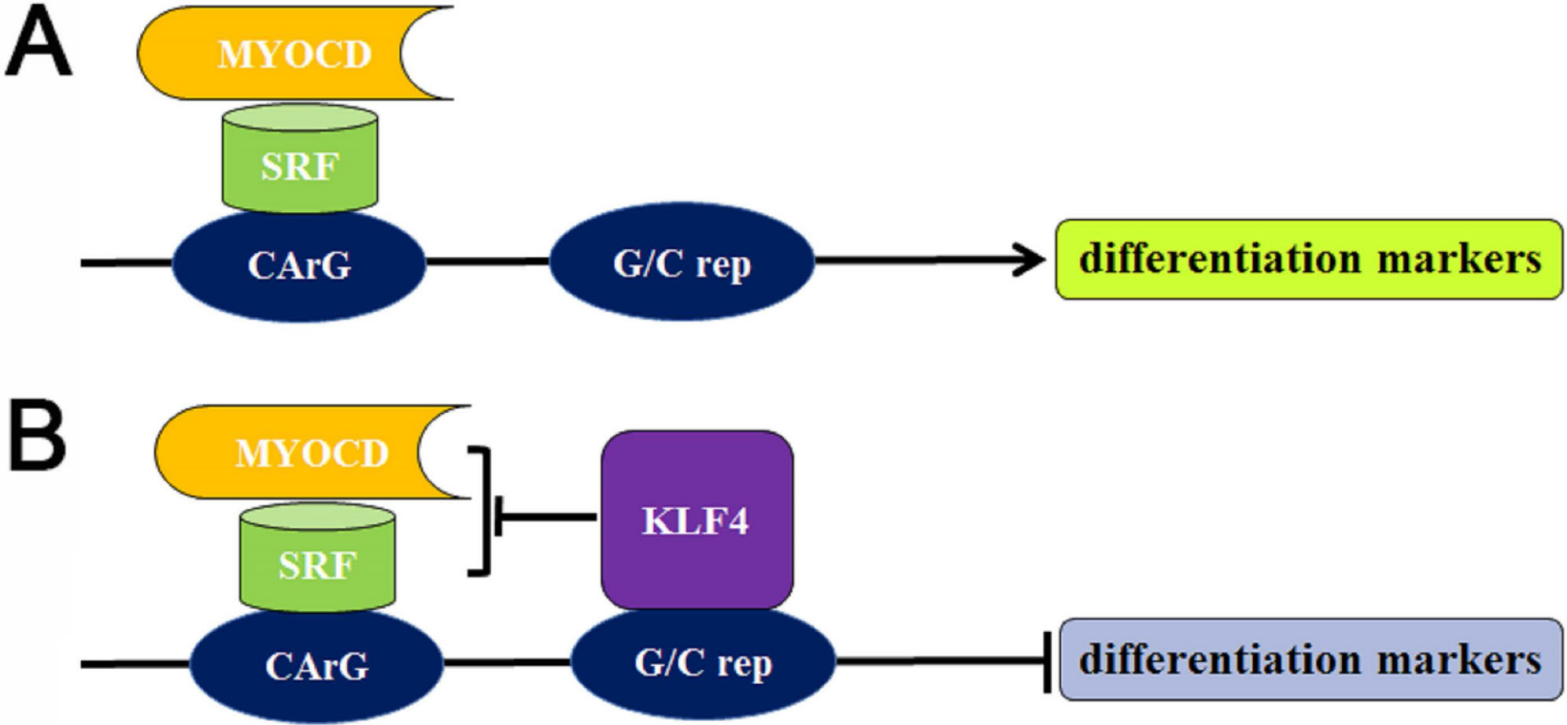
** Transcriptional regulation of the differentiation or de-differentiation of SMC markers. (A)** The MYOCD/SRF complex attaches to the CArG box for the expression of differentiation markers in contractile SMCs. **(B)** The absence of KLF4 in contractile SMCs, while increased level of KLF4 in synthetic SMCs. The binding of elevated KLF4 with G/C repressor suppresses the MYOCD/SRF complex, resulting in the transcriptional suppression of SMC differentiation markers. G/C rep: G/C repressor.

The different regulatory elements in SMC-specific cytoskeleton gene modulate the expression of these proteins. Myocardin (MYOCD) is a main protein that drives the formation of SMC contractile phenotype 23, 24, which forms a complex through combining with the ubiquitous serum response factor (SRF), followed by binding to the specific CArG sequence motif to promote the expression of differentiation markers. On the contrary, the pluripotent transcription factor Krüppel-like factor 4 (KLF4) is absent in contractile SMCs, which can promote the SMC dedifferentiation 25. KLF can not only bind to G/C repressor elements nearby CArG boxes, but also interact with SRF 26. KLF involves in the SMC dedifferentiation by inhibiting MYOCD expression, disrupting MYOCD/SRF complex, dissociating MYOCD/SRF complex, and modifying chromatin structure (**Figure 3**). In addition, other transcription factors, including phosphatase and tensin homolog (PTEN), phosphorylated ETS domain-containing protein-1 (Elk-1), Forkhead box protein O4 (FOXO4), Sp-1 and dedicator of Cytokinesis 2 (DOCK2), can also participate in the differentiation or de-differentiation of SMC via acting on the MYOD/SRF complex dynamics or interacting with KLF4 27.

In addition to gene modulation, the SMC phenotypes are also subject to programmed chromatin remodeling. Chromatin is constituted of nucleosomes, which is composed of 146 bp DNA wrapped around an octamer of histones. The histones are formed by two copies of histones H2A, H3B, H3 and H4. Chromatin exhibits in two different conformations including heterochromatin and euchromatin. The heterochromatin is a condensed DNA form, which can cause silencing of gene transcription, whereas the euchromatin is a non-condensed DNA form that can lead to the activation of gene transcription. Chromatin conformation is modulated by the acetylation of histone and methylation of lysine residues 28. In contractile SMCs, H3 and H4 relevant to CArG containing regulatory elements of α-SMA, SM22α, and SM-MHCs can be acetylated, leading to CArG elements bound by SRF. The increased activity of histone acetyltransferase (HAT) upregulates the expression of SM22α, while the elevated activity of histone deacetylase (HDACs) inhibits its expression 29. In addition, DNA demethylation also contributes to SMC differentiation. For instance, the DNA demethylase Ten-eleven translocation-2 (TET2) can oxidize 5-methylcytosis (5mC) to 5-hydroxymethycytosis, and therefore increase the DNA accessibility to transcription factors. In de-differentiated SMCs, the downregulation of TET2 is evidenced by both *in vitro* and in human coronary artery atherosclerotic plaque and experimentally induced intimal thickening. In cultured SMCs, TET2 knockdown reduced the accessibility of chromatin at the promoters of SMC contractile proteins, including α-SMA, MYOCD, and SMMHCs 29, 30. In a nutshell, SMC differentiation/dedifferentiation is regulated at both transcriptional and epigenetic levels.

## Role of SMCs in cardiovascular diseases

### SMCs in atherosclerosis

Atherosclerosis, a type of chronic progressive inflammatory disease, is a major cause of mortality worldwide. It is also a well-known process that lipid deposition occurred between the inner and middle layers of the blood vessel. This allows the macrophage infiltration and SMCs differentiation, and eventually leading to plaque formation 31. The major clinical consequences of this disease such as myocardial infraction or stroke are mainly caused by thrombotic events related to acute rupture or erosion of an unstable plaque, rather than gradual narrowing of vascular lumen 14. Historical observation in atherosclerosis showed that the migration and proliferation of SMCs within the intima allows the formation of initial atherosclerotic plaque, while they form fibrous caps to stabilize vulnerable plaques at the advanced stages. However, several studies on the genetic lineage tracing have suggested that the switching of SMC phenotype can not only lead to less differentiated forms in SMC 'markers' including macrophage-like cells, but also directly promote the development of atherosclerosis. Moreover, the proliferation of SMC may be beneficial for atherogenesis, however, the apoptosis of SMC, senescence, and SMC-derived macrophage-like cells may encourage atherosclerosis through inflammation.

Observation of atherosclerotic plaque composition in both animal models and human autopsies reveals that SMCs participate in the progression of atherosclerotic plaque at all stages, which include pre-atherosclerosis, early atherosclerosis, and late atherosclerosis 13. Since diffuse intimal thickenings (DITs) exist from birth, they are regarded pre-atherosclerotic plaques 32. Human DITs is composed of SMCs, proteoglycans and elastin, but lack of macrophages and thrombus. Most of SMCs that exhibit a synthetic phenotype in DITs are heterogeneous, as evidenced by a decreased genes encoding expression of contractile proteins and an increased ECM components generation. SMCs are considered as the major source of ECM in DITs, accounting for the increased intimal thickness, but, importantly for the development of atherosclerosis. DITs are full of proteoglycans, which are essential for the retaining apolipoproteins and subsequent inflammatory response.

ECM plays a crucial role in initiating atherosclerosis by the interaction between negative-charged proteoglycans and positive-charged apolipoproteins, which causes the retention of plasma-derived lipoproteins 33. The modifications of lipoproteins including LDL oxidation trigger the recruitment of macrophages and the initiation of the inflammatory response in atherosclerosis 34. During this process, the expression of contractile marker proteins, such as α-SMA, are attributed to both phenotypic switching and loss of SMCs through cell death including apoptosis, necrosis, and pyroptosis 34-36. For instance, the uptake of LDL oxidation and the formation of SMC-derived foam cells are associated with the induction of cell apoptosis 37, while the free cholesterol might be triggered by the dead SMCs 38. Therefore, SMCs play an essential role in initiation and early development of atherosclerosis.

Late atherosclerosis is featured by fibrous cap generation of SMCs and the formation of necrotic core, which are the consequences of efferocytosis defects of dead SMCs and macrophages 39. It is previously believed that intimal SMCs in the later stages of atherosclerosis are conducive to the formation of fibrous caps and protect plaques from rupture. Yet, the SMCs lipid loading and altered interactions with ECM cause the phenotype changes of SMC and increase macrophage markers expression in SMCs. Switching of SMCs to macrophage-maker-positive cells leads to a significant decrease in the thickness of intima and the cell content of α-SMA+ in the fibrous cap 40, 41. SMCs also play a critical role in the inflammatory milieu of the atherosclerotic plaque by recruiting macrophages. Further reports have strongly indicated that SMC-derived macrophage-like cells have direct impact on the progression of plaque. SMCs not only exhibit ECM-producing cells and macrophages of the fibrous cap, but also exhibit foam cells, mesenchymal stem cell (MSC)-like cells and osteochondrogenic cells in the plaque through phenotype switching 42, 43. Moreover, SMCs play a critical role in calcification of atherosclerotic plaques through apoptosis and osteochondrogenic conversion 44, 45.

During the course of atherosclerosis, the aberrant interplay between endothelial cells (ECs) and vascular SMCs (VSMCs) also play essential roles 46. For instance, the excessive turnover of VSMCs can be promoted by manipulating ECs in the formation of atherosclerotic plaque, while the EC injury-induced PDGF signaling is related to the proliferation of VSMC and the synthesis of ECM 47. In addition, the DNA demethylation of PDGF in ECs can activate VSMCs under homocysteine treatment 48. Moreover, ECs can suppress SMC proliferation and subsequent vascular injury through mediating vascular re-endothelialization through respectively inhibiting cell migration 49 and restenosis 50, enhancing the activity of peroxiredoxin 51, and inducing apoptosis of VSMCs 52.

According to these studies, an important target in the future is to identify and explore the potential factors and mechanisms that can result in the beneficial changes in atherosclerosis through mediating SMC phenotypes and functions, replacing these more conventional anti-atherosclerotic therapies.

### SMCs in hypertension

Hypertension, a leading risk factor of various cardiovascular diseases including stroke, coronary artery disease, peripheral vascular disease and heart failure, is related to vascular changes featured by endothelial dysfunction, increased vasoconstriction, and vascular remodeling 53. In hypertension, vascular remodeling involves changes to SMCs in the vessel wall, as well as endothelial cells, elastin and collagen contents. Among them, SMCs are critically involved in this process due to their plastic and dynamic features, as well as their ability of phenotypic switching. Pro-hypertensive stimuli, including oxidative stress, mechanical forces, renin-angiotensin-aldosterone system (RAAS), the activation of sympathetic nervous system and hemodynamic changes, can lead to vascular structural, functional and mechanical changes through stimulating SMC signaling 54-57.

As is known, the contraction and relaxation of SMCs quickly affects the diameter of blood vessels, which in turn affects blood flow velocity and changes the pressure on the blood vessel wall. The alteration of vascular diameter is an acute and rapid adaptive process, which is largely dependent on the activation and in-activation of the constrictor proteins in SMCs, namely the phosphorylation and dephosphorylation states. The contractile mechanisms of SMCs include actin and myosin as well as highly organized cytoskeleton 53. The cross-bridge formation of actin and myosin, actin filaments re-organization, intermediate filaments, and microtubules contribute to the vascular contraction. In SMCs, increased actin polymerization, activation of small GTP binding proteins, tyrosine phosphorylation of paxillin, and conformational changes in focal adhesion sites allow the stiffening and re-organization of the cytoskeleton. This dynamic rearrangement of the actin cytoskeleton is very important in maintaining vascular tone and plasticity, and regulating vascular diameter in hypertension.

In addition, SMC contraction in hypertension is also modulated by Ca^2+^-dependent and independent mechanisms. Many calcium-dependent mechanisms involved in regulating intracellular calcium homeostasis, including Ca^2+^ channels, contraction-related and unrelated signaling pathways, and immune and inflammatory systems. The perturbance of this homeostasis can cause abnormal vascular Ca^2+^ handling and high intracellular free calcium concentration ([Ca^2+^]_i_). In contrast, the calcium-independent processes can modify MLC sensitivity to Ca^2+^, and then regulate SMC contraction. Two major signaling pathways that associated with the calcium sensitization are DAG-PLC-PKC pathway and RhoA-Rho pathway kinase (ROCK) 58-61. Besides, other kinases such as p21-activated protein kinase, integrin-linked kinase (ILK), and zipper-interacting protein kinase (ZIPK) 62, 63, are also involved in this process. The calcium sensitization mechanism can regulate MLC20 phosphorylation independently of calcium-calmodulin-MLCK signaling.

Similar to calcium, reactive oxygen species (ROS) are also regarded as important second messenger molecules in SMCs. This is because the increased generation of vascular Nox-derived ROS in hypertension can result in increased concentration of intracellular free calcium and subsequently the rearrangement of cytoskeleton 64, 65, further causing the alteration of vascular reactivity and enhancement of contraction.

It is known that vascular contraction or vascular relaxation is modulated by calcium-dependent and calcium-independent manners. Emerging evidence indicated that the immune and inflammatory systems and the noncoding genome also contribute to the vascular dysfunction. Therefore, elucidating the complicated interactions between canonical pro-contractile calcium-regulated mechanisms and noncanonical pro-contractile mechanisms can provide better insights for the SMC phenotype switching in hypertension.

### SMCs in arterial aneurysm

Arterial aneurysm (AA) is a bulging in the wall of an artery, whose rupture is a catastrophe, including abdominal aortic aneurysm, thoracic aortic aneurysm, coronary artery aneurysm, and so on. In terms of morphology, arterial aneurysms are defined as local dilatation of the artery wall with partial loss of wall parallelism, while on the grounds of functions, arterial aneurysms are regarded as the gradual loss of the ability of the artery wall to withstand the wall tension caused by intracavity pressure, finally leading to acute rupture. Arterial aneurysm is correlated with arterial wall deformities and injury, as a result of infiltration of inflammatory cells, ECM degradation, and dysfunction of SMCs. Studies reported that infiltration of inflammatory cells, and synthesis and modelling of extracellular matrix are associated with SMCs 66, 67, indicating that SMCs play a very important role in the formation and progression of arterial aneurysms.

Previous studies elucidated that the excessive production of the proteases by SMCs, the phenotype switching of SMCs, and the loss of SMCs contribute to aneurysms of larger vessels, such as abdominal aortic aneurysm and thoracic aortic aneurysm. For example, Zhang et al. reported that Smad4-dependent TGF-β signaling in SMCs restrains the excessive production of the proteases essential for elastin degradation and protects against the development of aortic aneurysms. In addition, they demonstrated that Smad4 deficiency in SMCs directly triggers an inflammation-mediated progression of aortic aneurysms. These results indicated that Smad4 deficiency in SMCs promotes the development of aortic aneurysms through inducing the production of proteases and inflammation 68. As is known, a synthetic phenotype switched from a contractile phenotype in SMCs is considered as an important event in the development of abdominal aortic aneurysms. Peng et al. have demonstrated that VPO1 modulates the phenotypic switch of VSMC from contractile to synthetic via multiple signalling pathways including H_2_O_2_/VPO1/HOCl/ERK1/2 pathway. All of the above signaling pathways play a critical role in the development of abdominal aortic aneurysms 69. Li et al. substantiated that lncRNA H19 is capable of mediating the expression levels, cellular localization, and functionality of hypoxia-induced factor 1α (HIF1α), which exerts pro-apoptotic and anti-proliferation effects in aortic SMCs, resulting in subsequent abdominal aortic aneurysm-like dilation 70.

**Figure 4:**
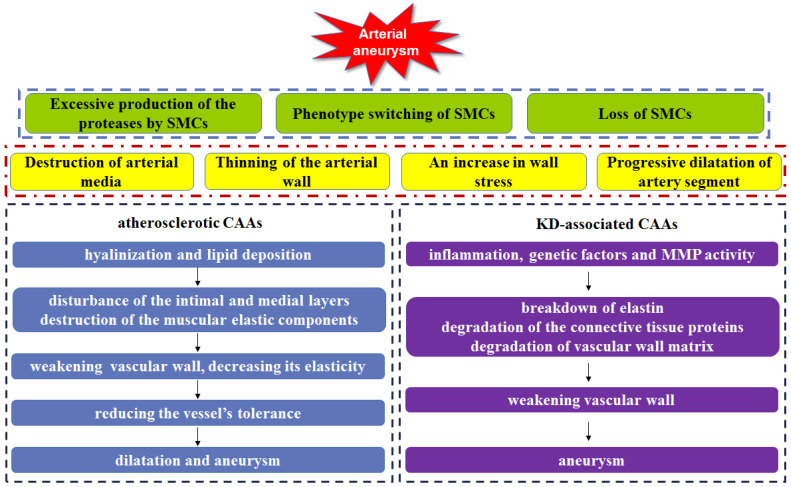
Arterial aneurysm can be induced by excessive production of the proteases by SMCs, phenotype switching of SMCs, and loss of SMCs, which lead to the arterial media destruction, the arterial wall thinning, an increase in wall stress and artery segment dilatation. Both hyalinization and lipid deposition in atherosclerotic CAAs result in the disturbance of the inner and medial layers in vessel wall, the muscular elastic components destruction, the vascular wall weakening and elasticity decreasing, allowing the reduction of vessel's tolerance, as well as vascular dilatation and aneurysm. In KD-associated CAAs, inflammation, genetic factors and MMPs activity lead to breakdown of elastin, degradation of the connective tissue proteins and vessel wall matrix, weakening vascular wall, finally inducing CAAs.

In addition to these aneurysms of larger vessels, coronary artery aneurysm is also a fatal cardiovascular disease, whose rupture will produce catastrophic effect in the patients. Coronary artery aneurysm (CAA) can cause a localized and irreversible dilatation of the blood vessel lumen, which increases the diameter of the adjacent normal segment by 1.5-fold 71. At present, although the pathophysiology is still not fully understood, CAA is similar to the aneurysm in larger vessels, characteristics by arterial media destruction, arterial wall thinning, wall stress increasing, and the dilatation of the coronary artery segment. Atherosclerosis is responsible for > 90% of CAAs in adults, on the other hand, Kawasaki disease is accountable for most cases in children 72 (**Figure 4**). Currently, the treatment options of coronary artery aneurysms remain unresolved.

Histological observation results of atherosclerotic CAAs showed that the hyalinization and lipid deposition can not only disturb the inner and medial layers of the vessel wall, but also the destruct muscular elastic components, which was associated with SMC phenotype switching 71, 73. In addition, several reports have also demonstrated the presence of calcification, fibrosis and large cholesterol crystals triggered the weakening of vessel wall and decreasing in elasticity. This caused further reduction of vessel tolerance to intraluminal pressures of blood flow, and thereby allowing the dilatation of vessels and the formation of aneurysms. Long-term transmural inflammation also contributes to the weakening of vessel walls. The over-stimulation of the vasodilator nitric oxide and the local mechanical stresses caused by stenosis may also induce the medial wall weakening of the coronary artery 74-76.

Kawasaki disease (KD), an acute inflammatory syndrome, is the most common causes of CAAs in children and the second in adults. KD may lead to acute vasculitis of the coronary arteries, which allows the subsequent dilatation of coronary artery and the formation of aneurysms. Accumulating studies have demonstrated that inflammation, genetic factors and matrix metalloproteinase activity contribute to the KD-associated CAAs. For instance, the elevated level of TNF-α is associated with the breakdown of elastin in KD patients 77, suggesting the vital role of inflammation in the progression of CAAs. Lin et al have clarified the close relationship between the genetic variant rs2833195 in the intron of the TIAM1 gene and the progression of CAAs in KD patients. TIAM1 protein may act a key role in chemokine-induced T cell migration, which allows the infiltration of lymphocytes into the vascular wall during the acute vasculitis stage of KD 78. Matrix metalloproteinase (MMP) is an enzyme responsible for degradation of the connective tissue proteins, and therefore weakening vascular wall. Aneurysmal vessels have shown elevated MMPs levels, whereas declined level of tissue-specific inhibitors of MMPs (TIMPs). This proteolytic imbalance drives the degradation of vessel wall matrix, subsequently leading to CAAs 79.

What's more, changes of SMC functions can also lead to CAAs. For example, Zhang et al reported that without miR-223 VSMC dedifferentiation proceeds undamped resulting in medial damage, apoptosis, and MMP9 release. The elevated MMP9 levels drive the degradation of arterial wall matrix, and subsequently promoting CAA formation 80-82. It is a well-known fact that SMCs senescence is also proven to enhance the formation and rupture of aneurysm as well 83, 84. Therefore, many different vascular SMC functions and intracellular signaling pathways are associated with the occurrence and progression of AA, which a defect in vascular SMC may be pathogenic. In addition, SMCs are unique to induce repair in the damaged vessel. To sum up, SMCs play a significant role in AA and become a potential target for further study.

### SMCs in MI

Acute myocardial infarction (MI) is myocardial necrosis induced by acute and persistent ischemia and hypoxia in the coronary arteries. Most of them occur on the basis of coronary atherosclerotic stenosis. Thrombus blocking coronary artery lumen can lead to myocardial ischemia and necrosis 85, which can also be induced by sharply increased myocardial oxygen consumption or coronary artery spasm. MI is a common cause that leads to elevated morbidity and mortality in patients with cardiovascular complications.

For the first time, Thidathip et al. analyzed transcripts of aortic vascular SMCs extracted by LCM from patients with MI and non-MI. Gene expression profiling revealed a new 21 gene SMCs-related classifier that distinguished the response of SMCs to MI at the transcriptome level. Vascular SMCs-related genes such as ATP1A2, MYOCD and SOD1 were found to be up-regulated in patients with acute MI 86. TATP1A2 encodes Na^+^/K^+^-ATPase that regulates the exchange of Na^+^ and K^+^ ions in response to the condition of metabolic stress, triggering increased vascular contraction of the thoracic aorta in mice 86. Myocardin (MYOCD) is a myogenic co-activator expressed in vascular SMCs. Previous study demonstrated that MYOCD interacts with SRF to induce gene expression in the CArG boxes, causing the differentiation of SMCs in mice 24. Superoxide dismutase 1 (SOD1) can regulate the level of H_2_O_2_ in the vasculature, thereby modulating the cellular oxidative stress of SMCs in mice 87. IPA analysis showed that in vascular SMCs of patients with MI, hypoxia signal of the cardiovascular system was the most important activation pathway, so the researchers also found the up-regulated HIF-1A, UBE2 and CREB1 genes that are associated with the hypoxia signaling pathway, while SOD1 of the aortic wall in MI patients was activated 71. The degradation pathway of superoxide radicals in protein analysis is abundant, in which the antioxidant enzymes SOD1 and CAT play a significant role. Meanwhile, the results of proteomics and transcriptome suggested that SMCs in the aortic wall activate the degradation pathway of superoxide free radical in patients with myocardial infarction. SOD1, an antioxidant that can protect cells from oxidative stress, can catalyze the conversion of superoxide anions into hydrogen peroxide and oxygen to reduce oxidative stress 88-89. Literature has shown that ROS production may be associated with the hypoxia and the level of electron transport chain cells in mitochondria 90. Hypoxia can cause an increase in the expression of SOD1. The production of ROS induces the remodeling of vascular wall by enhancing the MYOCD expression 91. In addition, the ROS can also regulate the expression level of cyclase-1- soluble A-3 (GUCY1A3), which is a risk gene of myocardial infarction 92.

Moreover, Guohong Ye et al. found that VSMCs secrete bFGF to the medium, and then activate the PI3K/AKT signaling pathway by binding to cardiomyocyte FGFR1 in the hypoxia state, thereby weakening the apoptosis and autophagy of myocardial I/R 93. STIM1 plays an important role in regulating calcium signaling pathways in different cell types, including SMCs 94. STIM1 detects changes in Ca^2+^ levels in the endoplasmic reticulum and interacts directly with Orai channels localized in the plasma membrane, coordinating to open Ca^2+^ channels (CRAC) activated by Ca^2+^ release 95. Specific absence of STIM1 in SMCs can significantly reduce the myocardial infarction area by decreasing ER stress, oxidative stress, inflammation and apoptosis. Furthermore, blocking STIM1 in SMCs protects the heart from chronic ischemia 96. Inactivation of the mineralocorticoid receptor (MR) in VSMCs has also been shown to improve left ventricular dysfunction and remodeling after myocardial infarction by maintaining coronary reserve and reducing oxidative stress-mediated coronary endothelial dysfunction 97. Harada et al reported that SMC sheet transplantation can significantly shorten fractional area, suppress left ventricular dilation, preserve wall thickness of the area at risk and the posterior wall, decrease fibrosis, and preserve myocardium in the scar area, eventually protecting cardiac function and minimizing cardiac remodeling in a rat MI model 98.

In brief, the above studies indicate that SMCs play a critical role in the process of MI. Through regulating SMC function or relevant SMC proteins, we can preserve the cardiac function under the background of MI.

## Conclusion

Cardiovascular disease is a major threat to human, especially for over 50 year-old patients. It is characterized by high morbidity, disability and mortality, so it is urgent to find the most advanced and perfect treatment. The research emphasized in this review contributes to our understanding that SMCs act as an essential role in the occurrence and development of cardiovascular diseases, such as atherosclerosis, hypertension, arterial aneurysms and myocardial infarction. Moreover, exploring its phenotypic changes, including contractile or synthetic phenotypes, and other cell types, such as macrophages, osteochondrogenic cells, mesenchymal stem cells and myofibroblast-like cells, provide a number of important implications for exploiting new targets and directions for clinical treatment. Although many successfully advances have been made in the study of SMCs, there is still much to be elucidated in this field. Therefore, the multifaceted roles of SMCs in cardiovascular disease should be further delineated so as to ensure to have a better understand of its mechanism, and obtain better therapeutic effects in clinical practices.
